# Comparison of Bone Density and Crestal Bone Loss Between Immediately Temporized and Submerged Implants in the Posterior Mandible: A Radiovisiographic Study

**DOI:** 10.1155/ijod/2797748

**Published:** 2025-09-16

**Authors:** Rodrigues Shobha, Prashant Bajantri, Gautami Pal, Sanchit Bansal, Arvind R., Srikant N., Mahesh M., Shetty Thilak, Pai Umesh, Saldanha Sharon, Sandipan Mukherjee, Ann Sales, Vignesh Kamath

**Affiliations:** ^1^Department of Prosthodontics, Manipal College of Dental Sciences Mangalore, Manipal Academy of Higher Education, Manipal 576104, Karnataka, India; ^2^Department of Prosthodontics, Kusum Devi Sunderlal Dugar Jain Dental College and Hospital, Kolkata 700002, West Bengal, India; ^3^Private Practitioner, Delhi, India; ^4^Department of Oral and Maxillofacial Surgery, Manipal College of Dental Sciences Mangalore, Manipal Academy of Higher Education, Manipal 576104, Karnataka, India; ^5^Department of Oral Pathology, Manipal College of Dental Sciences Mangalore, Manipal Academy of Higher Education, Manipal 576104, Karnataka, India

**Keywords:** bone density, provisionalized implant, radiovisiograph

## Abstract

**Purpose:** This study aimed to evaluate and compare alveolar bone density and crestal bone loss (CBL) around immediately temporized implants (ITIs) and conventionally placed submerged implants (CIs) in the posterior mandible using radiovisiography (RVG).

**Methods:** In this prospective, randomized, split-mouth clinical study, nine patients with bilateral posterior mandibular edentulism were enrolled. A bone-level implant was placed bilaterally in each patient. One side received an immediate temporized, while the contralateral side received a CI. Standardized RVGs were obtained at baseline, 3 months, and 6 months. Alveolar bone density and CBL were measured using ImageJ software. Data were analyzed using paired *t*-tests with significance set at *p* < 0.05.

**Results:** No statistically significant differences in alveolar bone density or CBL were observed between the ITI and conventional groups at any follow-up interval. Mean alveolar bone densities were higher in the ITI group at all intervals (baseline: 136.22 ± 36.23, 3 months: 137.03 ± 34, 6 months: 136.28 ± 34.59) compared to the conventional group (baseline: 136.56 ± 24.26, 3 months: 126.53 ± 34.93, 6 months: 119.95 ± 43.35). At 3 months, the ITI group exhibited greater CBL (0.22 ± 0.63 mm) than the conventional group (0.15 ± 0.83 mm). However, at 6 months, the ITI implants exhibited less CBL (− 0.07 ± 0.47 mm) compared to the conventional implants (0.19 ± 0.78 mm).

**Conclusion:** Immediate temporization under nonfunctional loading did not negatively affect peri-implant bone compared to conventional submerged healing. These results support its clinical use in appropriately selected cases.

## 1. Introduction

Dental implants have become a reliable and widely accepted treatment modality for the replacement of missing teeth, offering both functional and esthetic outcomes [1–3]. The long-term success of implant therapy is closely associated with the quality and quantity of alveolar bone [4]. Typically, the mandible exhibits thicker cortical bone and denser trabecular bone patterns compared to the maxilla [5–10]. Studies have shown that the anterior mandibular region has the highest bone quality, followed by the anterior maxilla, posterior maxilla, and posterior mandible, respectively, based on Hounsfield unit (HU) measurements obtained through computed tomography [8, 11]. Among the various bone-related parameters, bone density plays a critical role in determining implant stability, osseointegration potential, and the choice of surgical protocol. Denser bone offers greater resistance against occlusal and masticatory forces, making the evaluation of bone density essential from a clinical perspective.

Brånemark's traditional two-stage approach emphasized undisturbed healing for optimal osseointegration [12]. Clinical trials have reported high success and survival rates for implants that underwent submerged healing [13]. However, contemporary protocols support one-stage techniques, including immediate temporization, when sufficient primary stability is achieved. Immediate temporization, especially with nonfunctional loading, may accelerate rehabilitation, while preserving peri-implant tissues [14].

The one-stage procedure is recommended when sufficient primary stability (40 Ncm) is achieved during the surgical procedure by immediately temporizing the implant [15–17]. Temporized implants may be functionally or nonfunctionally loaded. In nonfunctional loading, the temporized crown has no occlusal contacts with its antagonist teeth in either centric or lateral positions, reducing the intensity of stresses on the implant [18–20].

Crestal bone loss (CBL) is a key parameter in evaluating implant success. Changes in bone density and marginal bone level are typically assessed radiographically. Studies have reported that during the first year of implant function, CBL in peri-implant tissue ranges from 0.9 to 1.6 mm, with the mean annual bone loss decreasing to 0.05–0.13 mm thereafter [14]. Concurrently, changes in alveolar bone density may reflect early biological responses and mechanical adaptation around implants. Following 1 year of loading, bone becomes denser, and occlusal forces that initiate CBL are insufficient to cause further bone loss. Initial demineralization may be perceived as CBL and reduced bone density. Demineralization can occur in crestal bone due to inflammation when hydroxyapatite (HA) is eliminated. Inflammation is associated with the overproduction of various cytokines and hyperactivation of osteoclasts, leading to bone loss. However, when inflammation subsides following adequate healing, particularly in clinical situations of implant placement, remineralization might be achieved by increasing osteoblast function. Osteoblasts promote crystal formation of HA, propagate growth in the interior part of membrane-limited matrix vesicles, and induce crystals in the collagenous extracellular matrix, thereby mineralizing the bone matrix overall.

Conventional intraoral periapical radiographs and panoramic radiographs have been used for presurgical evaluation and treatment planning but have limited value in detecting minimal changes in bone density. Digital subtraction radiography is also used for assessing bone density changes around an implant, but its sensitivity and specificity remain unanswerable [21]. Cone beam computed tomography (CBCT) is not reliable for bone density measurement due to distortion of HUs. It is also time-consuming (15–20 s), limited by its cost and availability and requires the patient to remain still during the scan [22].

In contrast, radiovisiography (RVG), combined with grayscale analysis through ImageJ software, offers less exposure and superior resolution, thereby providing a practical, noninvasive, and reproducible method for evaluating both bone density and marginal bone levels [23].

Despite numerous studies comparing loading protocols, few have concurrently explored both bone density and CBL using a split-mouth design in the posterior mandible. This study aims to fill this gap by evaluating and comparing bone density and CBL around immediately temporized and conventionally placed implants (CIs) over 6 months. The null hypothesis is that there will be no difference in bone density and CBL between immediately temporized implants (ITIs) and CIs.

## 2. Materials and Methods

### 2.1. Study Design and Ethical Approval

This study was designed as a prospective, randomized, split-mouth clinical trial and received approval from the Institutional Ethics Committee (Ref: IEC/16108). The study followed the CONSORT guidelines for reporting clinical research. All participants were informed about the nature of the study and provided written consent.

### 2.2. Participant Selection

Nine systemically healthy patients aged 20–50 years with bilateral edentulous spaces in the posterior mandible were recruited. All patients were nonsmokers, with no history of systemic illness or bone augmentation procedures. The inclusion and exclusion criteria are outlined in Table 1.

Each patient served as their own control, with one side randomly allocated to immediate temporization: Group A (ITI) and the other to conventional placement: Group B (CI).

### 2.3. Sample Size Calculation

The sample size was calculated based on previous literature and a study by Ramachandran A et al. [24–26], which reported standard deviations of 0.80 and 0.53 for grayscale value changes in functionally and nonfunctionally loaded implants, respectively. Using an average standard deviation (*σ*) of 0.665 and a minimum clinically relevant difference (*d*) of 0.9, with an alpha error of 5% and power of 80%, the required sample size was determined to be 18 implant sites, corresponding to nine patients treated bilaterally.

### 2.4. Surgical Protocol

CBCT was used for preoperative planning. Standardized diagnostic casts and stents were prepared. A single bone-level implant (MIS Seven, Confident India Ltd.) was placed by a single operator bilaterally in each patient using standard drilling protocols. Random allocation of implant type to each side of the mandible was performed using a left–right randomization method. The side allocated to Group A (ITI group) received immediate temporization with a nonfunctional acrylic provisional crown. The contralateral side, Group B (CI group), followed a conventional two-stage protocol with submerged healing.

### 2.5. Radiographic Assessment and Data Collection

Standardized RVGs were taken at baseline, 3 months, and 6 months using a paralleling technique with a grid and occlusal stent. All images were captured by the same operator using a consistent X-ray unit to minimize variability.

### 2.6. Bone Density and CBL Measurement (Figures 1-3)

Bone density was assessed using ImageJ software. A 30 × 30 pixel histogram tool was applied to measure mean grayscale values at six predetermined points around the implant (crestal, mid-crestal, and apical levels on both mesial and distal sides). The average of these values was taken as the bone density.

CBL was measured from the implant shoulder to the crestal bone level (DIB) on both mesial and distal sides, with values averaged per implant in millimeters (mm). CBL was defined as the change in DIB between the time intervals.

### 2.7. Statistical Analysis

Data were analyzed using Paired *t*-tests to compare bone density and CBL values between the ITI and CI groups at each time point. Statistical significance was set at *p* < 0.05.

## 3. Results

**Figure 4:**
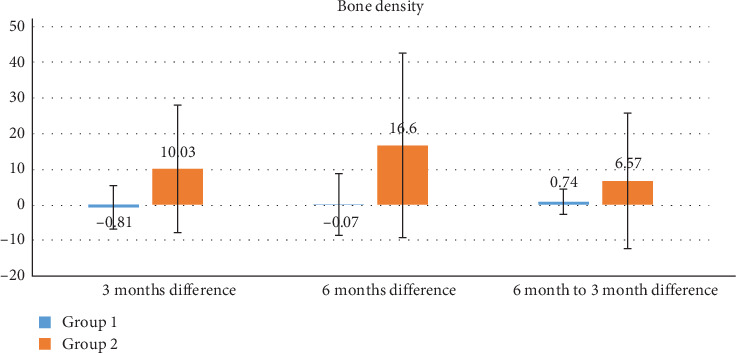
Graph representing the mean alveolar bone density difference of all groups at the different intervals of time.

**Figure 5:**
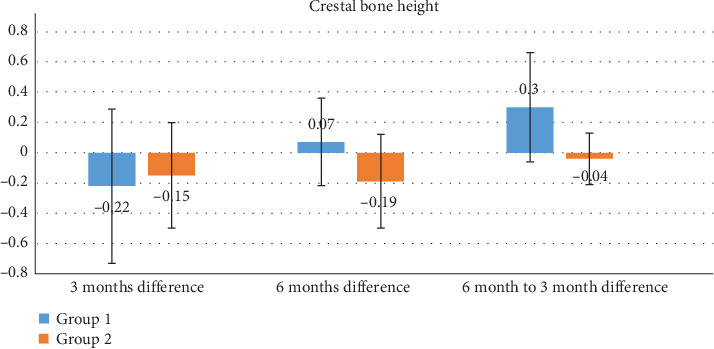
Graph representing the difference in crestal bone height at different time intervals in both groups. Group 1: immediately temporized implant; Group 2: conventional implant.

A total of nine patients completed the study protocol, each received one ITI and one CI, resulting in a total of 18 implants analyzed over a follow-up period of 6 months. (Tables 2 and 3; Figures 4 and 5). All implants osseointegrated successfully, with no signs of clinical mobility or infection.

### 3.1. Alveolar Bone Density

The mean alveolar bone density, measured in grayscale pixel values, for both the ITI and CI groups at baseline, 3 months, and 6 months, as well as the crestal bone height for the same time period, is presented in Table 2. While the ITI group consistently showed higher mean bone density values compared to the CI group across all time points, the differences were not statistically significant (*p* > 0.05). Specifically, the ITI group recorded values of 136.22 ± 36.23 at baseline, 137.03 ± 34.00 at 3 months, and 136.28 ± 34.59 at 6 months. In contrast, the CI group showed values of 136.56 ± 24.26 at baseline, which declined to 126.53 ± 34.93 at 3 months and further to 119.95 ± 43.35 at 6 months. Bone density values remained relatively stable across time in both groups, indicating favorable bone remodeling.

### 3.2. Crestal Bone Height

Crestal bone levels were compared across the three-time intervals.  Table 3 summarizes the results from paired *t*-tests. While a small increase in CBL was observed in the ITI group at 3 months (0.22 ± 0.63 mm), the CI group showed slightly lower values (0.15 ± 0.83 mm). By 6 months, the ITI group demonstrated a reduction in CBL (−0.07 ± 0.47 mm), whereas the CI group showed a net increase (0.19 ± 0.78 mm). These differences were not statistically significant.

## 4. Longitudinal Changes Within Groups

### 4.1. Bone Density

In ITI group, there were no statistically significant changes in mean bone density across any of the time points. The mean difference from baseline to 3 months was −0.81 ± 6.11 (*p*=0.701), from baseline to 6 months was −0.07 ± 8.69 (*p*=0.982), and from 3 to 6 months was 0.74 ± 3.62 (*p*=0.555). Similarly, in CI group, although there was a numerical reduction in bone density over time, these differences did not reach statistical significance. The mean difference from baseline to 3 months was 10.03 ± 17.95 (*p*=0.132), from baseline to 6 months was 16.6 ± 25.94 (*p*=0.091), and from 3 to 6 months was 6.57 ± 19.08 (*p*=0.332).

### 4.2. Bone Height

In ITI group, a statistically significant reduction in bone height was observed between 3 and 6 months, with a mean difference of 0.30 ± 0.36 mm (*p*=0.039). However, other comparisons in ITI group did not show significant changes: baseline to 3 months showed a mean difference of −0.22 ± 0.51 mm (*p*=0.226), and baseline to 6 months showed 0.07 ± 0.29 mm (*p*=0.464).

In CI group, none of the comparisons in bone height were statistically significant. The change from baseline to 3 months was −0.15 ± 0.35 mm (*p*=0.235), from baseline to 6 months was −0.19 ± 0.31 mm(*p*=0.102), and from 3 to 6 months was −0.04 ± 0.17 mm(*p*=0.502).

## 5. Graphical Summary

• Graph 1: Illustrates the mean alveolar bone density and CBL at all three intervals for both groups.
• Graph 2: Depicts differences in CBL, reinforcing the observed trend of greater stability in the ITI group.

## 6. Discussion

This prospective, randomized split-mouth study evaluated and compared alveolar bone density and CBL (CBL) around immediately temporized and CIs over a 6-month follow-up period using RVG and ImageJ software. The null hypotheses was accepted, since there was no statistical difference in the bone density and CBL between ITI and CI implants.

Both implant protocols demonstrated stable bone density over time, with the ITI group consistently showing higher mean grayscale values. Although these differences were not statistically significant, they may indicate enhanced osseous adaptation due to early functional stimulus or mechanical stimulation from soft tissues like the tongue and cheek, even under nonfunctional loading. Previous studies have similarly reported satisfactory osseointegration with immediate temporization under controlled loading conditions [27–29].

The trend of early CBL in both groups during the first 3 months aligns with prior findings that suggest bone remodeling is most active during the initial healing phase. The observed recovery in the ITI group, with reduced CBL at 6 months, supports the hypothesis that early temporization may promote favorable bone adaptation [30, 31]. In contrast, the CI group showed a continued mild increase in CBL.

CBL is influenced by multiple factors, including bone density, implant-abutment interface design, mucosal thickness, and mechanical stress. In the ITI group, slight additional forces from surrounding soft tissues and potential micromovements may have contributed to the early bone response. However, the absence of statistically significant differences suggests that these implants performed comparably within the short-term observation window.

The peri-implant environment undergoes dynamic remodeling influenced by mechanical loading, cellular activity, and inflammatory responses. According to Frost's mechanostat theory and Wolff's law, bone responds to mechanical strain by adapting its mass and density [32]. The ITI implants in this study may have benefited from early controlled stimuli, promoting osteoblastic activity without overloading the interface.

Using a split-mouth design eliminated interindividual variability and increased the study's power with a smaller sample size. Each patient served as their own control, improving comparability and reliability of outcomes [33, 34].

Large standard deviations observed, particularly in bone density values, could be attributed to individual variations in bone metabolism, oral hygiene, and healing response. These findings are consistent with previous literature indicating wide biological variability in peri-implant bone behavior [33, 34].

Despite promising findings, the study has several limitations. Limited sample size, short duration of the study and lack of histological validation make it difficult to extrapolate the results of the study to the larger population. Radiographic analysis, while standardized, lacks histological validation and may not fully capture microstructural changes. Future studies should include larger, more diverse populations, extend up to 12 months or beyond, explore additional outcome measures, such as implant survival rates, patient satisfaction, and peri-implant soft tissue response, compare functional vs. nonfunctional loading under immediate temporization protocols.

## 7. Clinical Relevance

Immediate temporization of dental implants, when carefully selected and performed under controlled nonfunctional loading conditions, can yield outcomes comparable to conventional two-stage placement in terms of bone density and CBL. This study supports the clinical utility of RVG and ImageJ software as practical, noninvasive tools for monitoring peri-implant bone changes. These tools provide clinicians with a reliable and standardized method for evaluating treatment success over time. These results may encourage clinicians to adopt immediate temporization strategies, improving patient comfort and reducing treatment duration without compromising clinical outcomes. However, larger-scale studies with long-term follow-up and histological correlation are warranted.

## 8. Conclusion

Within the limitations of this study, immediate temporization under nonfunctional loading showed no adverse impact on peri-implant bone when compared to conventional submerged healing. Both alveolar bone density and crestal bone stability were maintained over the 6-month period. These findings suggest that immediate temporization may be considered a clinically viable option in carefully selected cases.

## Figures and Tables

**Figure 1:**
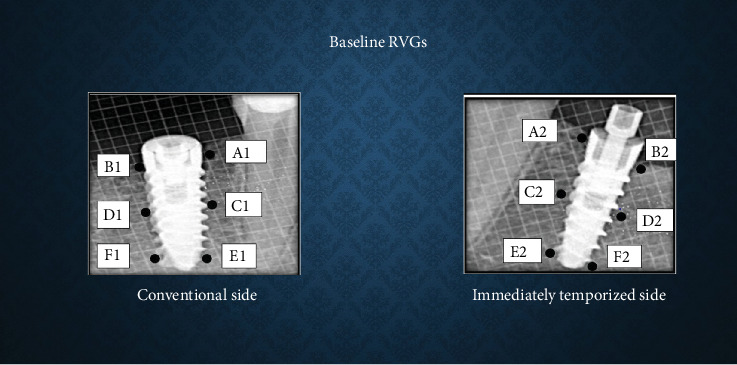
Bone density and crestal bone height measurement at baseline.

**Figure 2:**
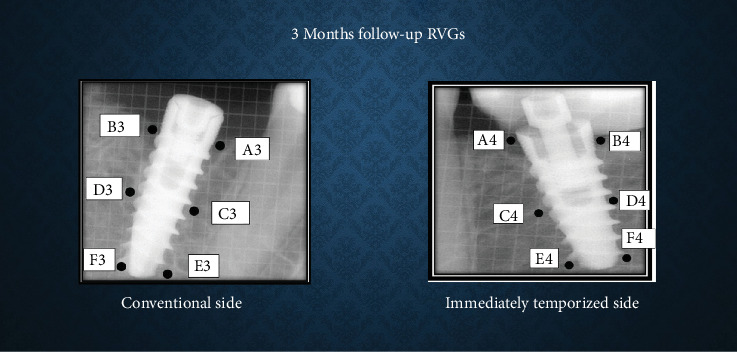
Bone density and crestal bone height measurement at 3 months' time interval.

**Figure 3:**
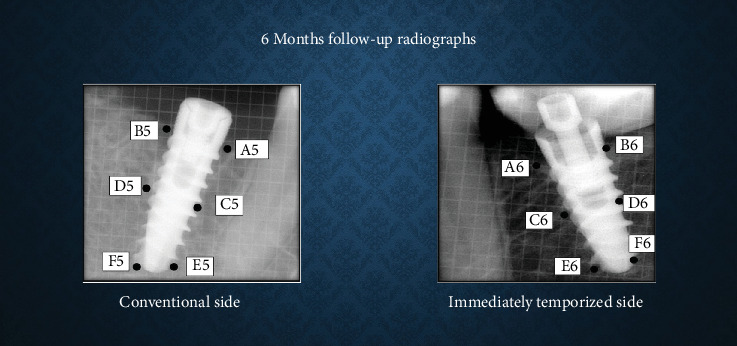
Bone density and crestal bone height measurement at 6 months' time interval.

**Table 1:** Inclusion and exclusion criteria followed in the study.

Inclusion criteria	Exclusion criteria
Partially edentulous area in either side of posterior mandible	Uncontrolled diabetes or other systemic diseases
At least one tooth missing in posterior mandible	Presence of defects at the desired site
Age limit 20–60 years	More than 60 years of age
Nondiabetic and nonsmokers	Smokers
No signs and symptoms of immunocompromised diseases	Patients with irregular follow-up
No bone or soft tissue augmentation procedure required during the surgery	Patients who cannot afford for the implant therapy
Antagonistic tooth present	With parafunctional habits

**Table 2:** Mean alveolar bone density (in gray scale pixels) and crestal bone height (in millimeters [mm]) at different intervals of time in both groups.

Parameter	Immediately temporized		Conventional		*t*	*p*-Value
	*N*	Mean ± sd	*N*	Mean ± sd		
Bone density baseline	9	136.22 ± 36.23	9	136.56 ± 24.26	−0.023	0.982
Bone density 3 months	9	137.03 ± 34	9	126.53 ± 34.93	0.646	0.527
Bone density 6 months	9	136.28 ± 34.59	9	119.95 ± 43.35	0.883	0.39
Bone height baseline	9	0.98 ± 0.35	9	0.99 ± 0.49	−0.045	0.965
Bone height 3 months	9	1.2 ± 0.52	9	1.14 ± 0.67	0.223	0.826
Bone height 6 months	9	0.91 ± 0.32	9	1.18 ± 0.61	−1.183	0.259

**Table 3:** Paired *t* test to see if bone density and crestal bone height changes over time.

		Bone density/crestal bone height	*N*	Mean ± SD	Mean difference ± SD	*t*	*p* Value
Group 1	Pair 1	Bone density baseline	9	136.22 ± 36.23	−0.81 ± 6.11	−0.40	0.701
		Bone density 3 months	9	137.03 ± 34			
	Pair 2	Bone density baseline	9	136.22 ± 36.23	−0.07 ± 8.69	−0.02	0.982
		Bone density 6 months	9	136.28 ± 34.59			
	Pair 3	Bone density 3 months	9	137.03 ± 34	0.74 ± 3.62	0.62	0.555
		Bone density 6 months	9	136.28 ± 34.59			
	Pair 4	Crestal bone height baseline	9	0.98 ± 0.35	−0.22 ± 0.51	−1.31	0.226
		Crestal bone height 3 months	9	1.2 ± 0.52			
	Pair 5	Crestal bone height baseline	9	0.98 ± 0.35	0.07 ± 0.29	0.77	0.464
		Crestal bone height 6 months	9	0.91 ± 0.32			
	Pair 6	Crestal bone height 3 months	9	1.2 ± 0.52	0.3 ± 0.36	2.47	**0.039**
		Crestal bone height 6 months	9	0.91 ± 0.32			

Group 2	Pair 1	Bone density baseline	9	136.56 ± 24.26	10.03 ± 17.95	1.68	0.132
		Bone density 3 months	9	126.53 ± 34.93			
	Pair 2	Bone density baseline	9	136.56 ± 24.26	16.6 ± 25.94	1.92	0.091
		Bone density 6 months	9	119.95 ± 43.35			
	Pair 3	Bone density 3 months	9	126.53 ± 34.93	6.57 ± 19.08	1.03	0.332
		Bone density 6 months	9	119.95 ± 43.35			
	Pair 4	Crestal bone height baseline	9	0.99 ± 0.49	−0.15 ± 0.35	−1.28	0.235
		Crestal bone height 3 months	9	1.14 ± 0.67			
	Pair 5	Crestal bone height baseline	9	0.99 ± 0.49	−0.19 ± 0.31	−1.85	0.102
		Crestal bone height 6 months	9	1.18 ± 0.61			
	Pair 6	Crestal bone height 3 months	9	1.14 ± 0.67	−0.04 ± 0.17	−0.70	0.502
		Crestal bone height 6 months	9	1.18 ± 0.61			

*Note:* Significance is set at *p* < 0.05. The difference in crestal bone height betweeen 6 and 3 months is significant therefore in bold.

## Data Availability

The data that support the findings of this study are available upon request from the corresponding author. The data are not publicly available due to privacy or ethical restrictions.
